# Malaria in Hadhramout, a southeast province of Yemen: prevalence, risk factors, knowledge, attitude and practices (KAPs)

**DOI:** 10.1186/1756-3305-7-351

**Published:** 2014-07-29

**Authors:** Omar AA Bamaga, Mohammed AK Mahdy, Rohela Mahmud, Yvonne AL Lim

**Affiliations:** Department of Parasitology, Faculty of Medicine, University of Malaya, 50603 Kuala Lumpur, Malaysia; Research Department, University of Science and Technology, Taiz, Yemen; Department of Parasitology, Faculty of Medicine, Sana’a University, Sana’a, Yemen

**Keywords:** Malaria, *Plasmodium*, Epidemiology, KAP, Hadhramout, Yemen

## Abstract

**Background:**

Yemen is a Mediterranean country where 65% of its population is at risk of malaria, with 43% at high risk. Yemen is still in the control phase without sustainable reduction in the proportion of malaria cases. A cross-sectional household survey was carried out in different districts in the southeast of the country to determine malaria prevalence and identify factors that impede progress of the elimination phase.

**Methods:**

Blood specimens were collected from 735 individuals aged 1–66 years. *Plasmodium* species were detected and identified by microscopic examination of Giemsa-stained thick and thin blood smears. A household-based questionnaire was used to collect demographic, socioeconomic and environmental data.

**Results:**

The overall prevalence of malaria was 18.8% with *Plasmodium falciparum* as the predominant species (99.3%), with a low rate of *Plasmodium vivax* detected (0.7%). The infection rate was higher in Al-Raydah and Qusyer districts (21.8%) compared to Hajer district (11.8%). Fifty-two percent of the persons positive for *Plasmodium* were asymptomatic with low parasite density. The adults had a higher infection rate as compared to children. Univariate analysis identified those whose household’s head are fishermen (OR = 11.3, 95% CI: 3.13 – 40.5) and farmers (OR = 4.84, 95% CI: 1.73 – 13.6) as high-risk groups. A higher number of positive smears were observed in people living in houses with uncemented brick walls (OR = 2.1, 95% CI: 1.32 – 3.30), without access to toilets (OR = 1.6, 95% CI: 1.05 – 2.32), without a fridge (OR = 1. 6, 95% CI: 1.05 – 2.30), or without TV (OR = 1. 6, (95% CI: 1.05 – 2.30). People living in houses with water collection points located less than 200 meters away were also at higher risk of acquiring malaria (OR = 1.6, 95% CI: 1.05 – 2.30). Knowledge about the importance of using insecticide-treated mosquito nets (ITNs) and indoor residual spraying (IRS) for prevention of malaria was 7% and 2%, respectively.

**Conclusions:**

Several environmental, socioeconomic and behavioral issues were discovered to be the contributing factors to the high prevalence of malaria in southeast Yemen. Novel strategies adapted to the local situations need to be established in order to improve the effectiveness of malaria control.

## Background

Malaria is a major health problem worldwide with 3.3 billion individuals at risk leading to morbidity and mortality, especially among children under five years of age and pregnant women [1–3]. In the Eastern Mediterranean region, which consists of 10 countries including Yemen, 300 million people are at risk of malaria [3].

A majority of the Yemeni population (i.e., 65%) is exposed to malaria transmission, with 43% being at high-risk of acquiring the infection [3, 4]. *Plasmodium falciparum*, the most dangerous species is the major *Plasmodium* species in Yemen with only minimal cases caused by *Plasmodium vivax* [5]. Although *Anopheles arabiensis* has been reported as the main vector within the country, *Anopheles culicifacies* is an important vector in the coastal areas, and *Anopheles sergenti* has been reported to be a vector in the mountainous hinterland and highland areas [3, 6]. Following the emergence of chloroquine resistance and the WHO recommendation to withdraw artemisinin-based monotherapies, the antimalarial treatment policy shifted to artemisinin-based combination therapy (ACT) with artesunate + sulphadoxine-pyrimethamine (SP) as the first-line, and artemether-lumefantrine (AL) as the second line therapy for uncomplicated malaria [3]. However, chloroquine is still being used by clinicians in both public and private health facilities because of the limited and poor knowledge of the newer treatment policy [7].

The National Control Malaria Program (NCMP), Yemen, is proactive in combating malaria through the implementation of several interventions that include distribution of insecticide-treated mosquito nets (ITNs), indoor residual spraying (IRS), proper diagnosis, proper treatment, and reactive and proactive case surveillance. However, Yemen is not on track to achieve the Global Malaria Action Plan (GMAP)’s objective, which is to reduce global malaria cases by 75% by the end of 2015 [8]. Previous studies showed high prevalence of malaria in Yemen with mortality rates ranging from 2.1 – 4.7% in children [4–6, 9, 10]. Although Yemen has been classified as being in the control phase, the 2013 World Malaria Report stated that the data (collected in 2011) on which the report is based were insufficient to estimate the trend of malaria case incidence. By contrast, Saudi Arabia, the northern neighbouring country of Yemen, showed more than 75% reduction in malaria case incidences placing it in the elimination phase, and Oman, the eastern neighbouring country of Yemen is now in the prevention of re-introduction phase [3, 8, 11].

In light of the current malaria situation, the present study aimed to determine the prevalence and risk factors of malaria in the southeast of Yemen, and to explore the residents’ knowledge, attitude and practices (KAP) toward malaria. It is hoped the findings from this study could assist in identifying factors that impede progress to the elimination phase.

## Methods

### Study areas and study population

**Figure 1:**
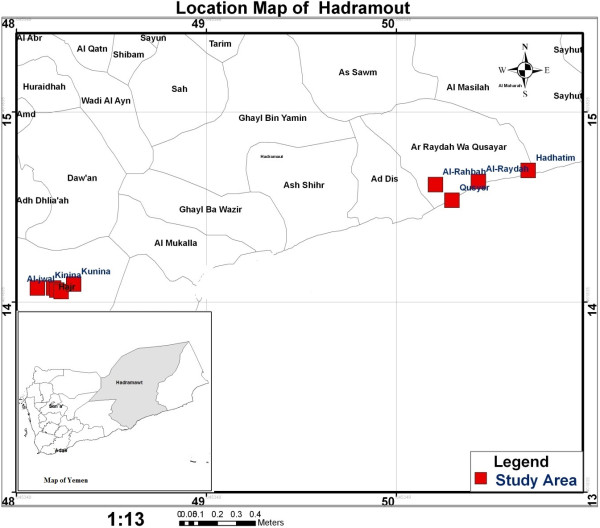
**A geographic map of the study area.**

The study was conducted in the Hadhramout governorate in the south-east Yemen, the largest governorate, accounting for half of the country’s surface area. The population of this governorate was estimated at 1,028,556 [12]. Malaria transmission in Yemen differs between the regions. In the coastal areas, peak transmission occurs in winter (October - April), which is the case in Hadhramout, while in the mountainous hinterland areas it usually peaks in summer (May - September). However, in highland areas located above 2000 meters above sea-level, transmission occurs throughout the year [13]. The sources of income of the population under study include funds transferred from natives working in neighbouring Gulf countries, agriculture, fishing, livestock or handicraft. A total of 735 participants of all ages and genders were enrolled in this study; 221 participants from three villages in Hajer district and 514 participants from four villages in Al-Raydah-Qusyer district (Figure 1). Informed consent was obtained from each participant, and for children, consent was obtained from their parents after a clear explanation of the study objectives. The study protocol was approved by the Faculty of Medicine, Hadhramout University for Science and Technology, the Ministry of Health and Population, Yemen, and the Malaria National Control Program division in Hadhramout governorate.

### Samples

A household survey was conducted by surveyors who had previous experience with malaria surveys. Households were randomly selected and all household members were invited to participate. Data were collected during transmission seasons from July 2011 to May 2012. Blood samples were collected by the finger prick method and thin and thick blood smears were made, allowed to air-dry (the thin smears were fixed with methanol within three hours), and then brought back to the laboratory to be stained with Giemsa. Haemoglobin levels were measured in the field directly from capillary blood using the HemoCue haemoglobinometer (HemoCue, AB, Angelhom, Sweden). Coordinates of each village was recorded using a global positioning system (GPS) (Garmin GPSMAP 60CSx, Tonopah, AZ, USA).

### Questionnaire

A pretested standard questionnaire was used to collect information about personal profile, socioeconomic, and environmental background. A checklist was used for malaria clinical signs and symptoms, as observed by a team of physicians, and any history of previous antimalarial treatment. Knowledge, attitude and practices (KAP) were investigated using a standard questionnaire. The data were collected from the household members, or from the parents on behalf of children, via face-to-face interviews conducted by well-trained interviewers. During the interviews, direct observation was made for the type of household building, wall, floor; for the availability and the type of toilet facilities, piped water, clothes-wearing habits, electricity, telephone, mosquito nets, and finally in the presence of nearby pools or rivers. The signs and symptoms recorded included fever and jaundice. Parasitaemia was expressed as the total number of *Plasmodium* asexual forms per microliter of blood. Parasite levels were classified as low (1 - 999/μL), moderate (1000 - 9999/μL), or high (>10000/μL). Hemoglobin levels were considered as normal (>11 g/dl), low anaemia (9–11 g/dl), moderate anaemia (7–8.9 g/dl), and severe anaemia (<7 g/dl) [14].

### Microscopic examination

Blood films were stained with 10% Giemsa stain and examined by three trained malaria microscopists following standard procedures. Species identification was performed in the laboratory of the National Malaria Control Program in Hadhramout governorate by three expert microscopists. Parasitaemia per μl of blood was calculated from thick smears by counting the number of asexual parasites per 200 leukocytes using an assumed leukocyte count of 8000 WBC/μl. A smear was recorded as negative after screening at least 100 high power microscope fields.

### Statistical analysis

Data analysis was performed using *the Statistical Package for Social Sciences for Windows* (SPSS) version 19.0. The significance of the associations and between proportions of variables was tested using the chi-square and Fisher exact tests. A significance level of 0.05 at 95% confidence interval (CI) and odd ratios (OR) were computed. A stepwise conditional logistic regression model was developed for those variables with p value <0.05. The significance level was considered as P < 0.05.

## Results

### Characteristic of study population

A total of 735 people voluntarily participated in this study, 423 males (42.4%) and 312 (57.6%) females. Forty-seven percent of the study population had no formal education and 61.6% were farmers. Approximately half of the study population had no access to electricity or communication media such as radio or television. The study population lived in simple houses with mud or uncemented brick walls and mud or cement floors (Table 1).

**Table 1:** **Characteristics of study subjects**

Characteristics	Number (%)
**Gender**	
Male	423 (42.4)
Female	312 (57.6)
**Age (years)**	
>15	393 (53.5)
10 – 15	142 (19.3)
5 - 9	152 (20.7)
<5	48 (6.5)
**Districts**	
Hajer	221 (30.1)
Al-Raydah and Qusyer	514 (69.9)
**Family size**	
>5 members	290 (39.5)
≤5 members	445 (60.5)
**Education**	
Not educated	345 (46.9)
Primary	356 (48.4)
Secondary	34 (4.6)
**Occupation**	
Not working	180 (24.5)
Farmer	453 (61.6)
Fisherman	26 (3.5)
Government employees	76 (10.3)
**Economic status**	
Houses with electricity	379 (51.6)
Availability of TV	295 (40.1)
Availability of telephone	43 (5.9)
Availability of radio	385 (52.4)
Availability of fridge	295 (40.1)
Having motorcycle	148 (20.1)
Having car	212 (28.8)

### Prevalence and factors associated with malaria

The overall malaria prevalence in the persons sampled in the Hadhramout governorate of Yemen was 18.8%. *Plasmodium falciparum* was the predominant species (99.3%) followed by *P. vivax* (0.7%). The overall prevalence in the districts of Al-Raydah and Qusyer and of Hajer were 21.8% and 11.8%, respectively. However, broad variation in the prevalence was equally noted between the villages from both districts, with Qusyer showing the highest prevalence (31.8%) and Al-Raydah the lowest (5.6%) (Table 2).

**Table 2:** **Prevalence and distribution of malaria stratified by areas**

Characteristics	Examined	Infected (%)	P value
**Districts**			
Hajer	221	26 (11.8)	0.001
Al-Raydah and Qusyer	514	112 (21.8)	
**Hajer District Villages**			
Kunina	83	5 (6)	0.001
Kinina	47	12 (25.5)	
Jol-Bamejah	91	9 (9.9)	
**Al-Raydah and Qusyer District Villages**			
Hadhathim	34	10 (29.4)	
Al-Raydah	18	1 (5.6)	
Qusyer	22	7 (31.8)	
Al-Rahbah	440	94 (21.4)	

The number of parasite positive individuals was higher in men than in children and women. Persons whose household’s head had primary education were at higher risk of being infected (OR = 10.1, 95% CI: 1.35 – 74.5), as did fishermen (OR = 11.3, 95%CI: 3.13 – 40.5) and farmers (OR = 4.84, 95%CI: 1.73 – 13.6). A number of socio-economic indicators were also associated with increased prevalence: living in houses with walls made of uncemented bricks (OR = 2.1, 95% CI: 1.32 – 3.30), no access to toilets (OR = 1.6, 95%CI: 1.05 – 2.32), no fridge (OR = 1.6, 95%CI: 1.05 – 2.30), or no TV (OR = 1.6, (95%CI: 1.05 – 2.30). People living in houses with distance of water collection points less than 200 meters were also at higher risk of acquiring malaria (OR = 1.6, 95%CI: 1.05 – 2.30) (Table 3). Multivariate analysis using stepwise forward logistic regression confirmed that the significant risk factors were living in uncemented brick wall houses and being a fisherman or a farmer.

**Table 3:** **Factors associated with malaria in Hadhramout governorate of Yemen**

Characteristics	Examined	Infected (%)	OR (95%CI)
**Age (years)**			
>15	393	79 (20)	1
10 – 15	142	25 (17.6)	0.85 (0.51 – 1.40)
5 - 9	152	30 (19.7)	0.98 (0.61 – 1.56)
<5	48	4 (8.3)	0.36 (0.13 – 1.04)
**Gender**			
Female	312	52 (16.7)	1
Male	423	86 (20.3)	1.04 (0.98 – 1.12)
**Education level household’s head**			
Secondary school & above	34	1 (2.9)	1
Primary school	356	83 (23.3)	10.1 (1.35 – 74.5)
Not educated	345	54 (15.7)	6.12 (0.82 – 45.7)
Occupation of household’s head*			
Government employees	76	4 (5.3)	1
Not working	180	28 (15.6)	3.31 (1.12 – 9.80)
Farmer	453	96 (21.2)	4.84 (1.73 – 13.6)
Fisherman	26	10 (38.5)	11.3 (3.13 – 40.5)
**Family size**			
>5 members	290	49 (16.9)	1
≤5 members	445	89 (20)	1.23 (0.84 – 1.81)
House wall*			
Mud	221	26 (11.8)	1
Uncemented bricks	514	112 (21.8)	2.1 (1.32 – 3.30)
**Material of house floor**			
Cement	120	19 (15.8)	1
Mud	615	119 (19.3)	1.27 (0.75 – 2.16)
**Availability of toilet**			
Yes	284	42 (14.8)	1
No	451	96 (21.3)	1.6 (1.05 – 2.32)
**Distance to the nearest water collection**			
> 200 meters	295	44 (14.9)	1
≤ 200 meters	440	146 (18.6)	1.6 (1.05 – 2.30)
**Availability of electricity**			
Yes	379	66 (17.4)	1
No	356	72 (20.2)	1.04 (0.97 – 1.11)
**Availability of fridge**			
Yes	295	44 (14.9)	1
No	440	94 (21.4)	1.6 (1.05 – 2.30)
**Availability of TV**			
Yes	295	44 (14.9)	1
No	440	94 (21.4)	1.6 (1.05 – 2.30)
**Availability of radio**			
Yes	385	70 (18.2)	1
No	350	68 (19.4)	1.02 (0.95 – 1.09)
**Availability of telephone**			
Yes	43	8 (18.6)	1
No	692	130 (18.8)	1.0 (0.87 – 1.16)

*Variables confirmed as significant factors associated with malaria using stepwise forward logistic regression.

### Knowledge, Attitude and Practices (KAP)

The survey of the villagers’ knowledge, attitude and practices toward malaria indicated that although they are all aware of malaria, its mode of transmission, and its clinical symptoms and severity, their knowledge of and attitude towards malaria prevention were poor. Thus, only 7% and 2% of study participants mentioned the importance of sleeping under insecticide-treated mosquito nets (ITNs) or using indoor residual spraying (IRS) as methods of malaria prevention, respectively. This concorded with a low usage of ITNs (8%). Furthermore, in all cases, the windows in the houses were kept open at night (Table 4).

**Table 4:** Knowledge, Attitude and Practices (KAPs) of study subjects with regards to malaria in the rural areas of Southeast of Yemen (n = 130)*

Characteristics	Number (%)
*Knowledge and attitudes*	
**Know malaria**	130 (100)
**Malaria can kill**	53 (51)
**Mode of transmission mentioned**	
Mosquito bite	130 (100)
Lack of sanitation	4 (3)
Swamps	19 (15)
**Causes of malaria mentioned**	
Flies	42 (32)
Sleeping with infected person in the same bed	106 (82)
Mosquito bite	130 (100)
Drinking or playing in contaminated water	0 (00)
The presence of sewage	9(7)
**Symptoms of malaria mentioned**	
Fever	53 (41)
Fever + shivering	77 (59)
**Serious for adult or children**	
Children	118 (91)
Equally serious	12 (9)
**Methods of prevention mentioned**	
Cleaning the house or environment	118 (91)
Sleeping under the mosquito net	9 (7)
House spraying with insecticides	3 (2)
Smoking house	69 (53)
*Practices*	
Using insecticide-treated mosquito nets (ITNs)^a^	14 (11)
House spray with insecticide (IRS)^#^	130 (100)
Not closing house windows	130 (100)
Closing house doors	130 (100)
Going to clinic when having fever	22 (17)
Houses with wood roofs	130 (100)
Houses with uncemented bricks wall	93 (72)
Houses with mud wall	37 (28)
Keeping uncovered water near houses	130 (100)

*KAPs were conducted on the head of the household.

#IRS was done by government before one year of the survey.

^a^Each house of the 14 houses had one ITNs.

### Clinical manifestations

More than half of malaria cases detected by microscopy were asymptomatic. The symptomatic cases presented with fever, shivering, jaundice and anaemia. The parasitaemias were recorded as low, moderate and severe in 52%, 35% and 13% of malaria cases, respectively. A positive association between clinical symptoms and parasitaemia was observed (χ^2^ = 422, p <0.001) (Table 5).

**Table 5:** **Clinical manifestations of malaria cases (N = 138)**

Characteristics	Prevalence N(%)	P value
Presence of fever*		
Yes	66 (48)	<0.05
No	72 (52)	
Presence of shivering*		
Yes	38 (27.5)	<0.05
No	100 (72.5)	
Presence of headache*		
Yes	21 (15)	<0.05
No	117 (85)	
Presence of jaundice*		
Yes	14(10)	<0.05
No	124 (90)	
**Haemoglobin level**		
Normal	13 (9)	<0.05
Low anaemia	92 (67)	
Moderate anaemia	33 (24)	
**Total**	138	

*Fisher exact test was used.

## Discussion

Although Yemen is classified as in the control stage [15], Hadhramout governorate, located in the southeast of the country bordering Oman and Saudi Arabia, is considered to be in the pre-elimination phase and a bilateral collaboration between Yemen and Oman has been put in place with the aim of making this a malaria-free area (personnel communication). The purpose of the current survey was to evaluate the actual status of malaria in the Hadhramout community and to investigate factors that might challenge or slow the progress toward malaria elimination.

The overall microscopic prevalence recorded for malaria in the 735 persons sampled from the seven sites was 18.8%. These high values are inconsistent with a pre-elimination status, and rather placed this governorate in the control phase. Moreover, the prevalence of malaria in young children (2–9 years old) exceeds the 10% level indicative of high to moderate transmission [16]. This high prevalence could be attributed to several factors including the political instability in Yemen during the 2011 – 2012 period, which had a direct effect on the official programs to control and to combat malaria. It was noted that the last IRS was conducted one year before the field trip. It is also likely that new foci of malaria have emerged in this area, which had been considered of low endemicity. Although the prevalence of malaria cases is decreasing in Hajer district [17], in the traditional malaria endemic area in Hadhramout, an increase of prevalence was recorded in Al-Raydah and Qusyer districts, areas thought to be of low prevalence. This situation poses a challenge to control efforts.

Analyses of the data from the survey presented here have identified some factors that were associated to the increased risk of acquiring malaria. These factors should be taken into consideration when implementing future malaria control strategies. Thus, malaria was more prevalent in adults than in children, who generally constitute the high-risk group. Multivariate analysis confirmed that people whose household’s head are fishermen and farmers were at higher risk of being malaria positive. It should be mentioned that household members in Yemen actively contribute to the work of the head of the household. Such increased risk of malaria linked to occupational behavior has been noted in other endemic areas such as Malaysia [18], the Philippines [19] and Latin America [20, 21]. These observations indicated that exposure to the bite of infective mosquitoes occured outside the home. Consequently, the traditional vector control interventions (ITNs and IRS) that protect household members would be insufficient, and control measures should be implemented to reduce mosquito-human contact during outdoor activities. Occupation-based vector control interventions have been developed and have shown reduction in malaria cases in Pakistan [22], Afghanistan [23] and Vietnam [24]. Such interventions include topical repellents such as N, N-diethyl-3-methylbenzamide (DEET) [25], DEET-based soap [22], plant based repellant [26], long-lasting insecticide-treated hammocks for forest workers [27] and insecticide-treated personal clothes in refugee areas [23].

Nonetheless, transmission in and around the house remains significant, as indicated by the statistical analyses which showed that the type of housing, unavailability of in-house toilets, and the presence of uncovered water containers close to the houses are also significant predictors of malaria in Hadhramout. Thus, it will be important to improve the environment and economic status of the inhabitants if the government’s efforts to make Hadhramout free of malaria are to be fulfilled.

At present, the malaria control strategy in Yemen relies on the adequate distribution of and use of ITNs, as well as the deployment of the IRS as the main intervention for vector control. It is, therefore, of concern that in the present survey only 7% of the people expressed the belief that sleeping under ITNs protected them from malaria (only 8% actually indicated that they sleep under ITNs) and only 2% considered that IRS protects them from malaria. This unsatisfactory situation is not unique to this district, as a malaria indicator survey conducted in Yemen in 2008–2009 revealed that 4.2% of people and 7% of children under 5 years slept under long lasting insecticide-treated net (LLINs) [3]. This represents a major challenge that warrants an urgent action. Finally, the fact that more than half of malaria positive persons identified in the current study were asymptomatic with low parasite densities suggests that it is likely that these cases would be missed by passive surveillance and would thus remain as a source of malaria transmission [28, 29].

## Conclusion

In conclusion, malaria remains an important public health concern in the southeast region of Yemen, where there seems to be an upward shift in malaria prevalence with the appearance of new endemic foci and occupational high-risk groups. This warrants innovative strategies that should focus on reducing both indoor and outdoor transmissions in order to achieve effective protection from malaria. Furthermore, active case detection (ACD) should be implemented as this would be crucial to identify and treat the substantial reservoir of asymptomatic persons in the community. In particular, the reversal of the perplexing low awareness of the importance of ITNs and IRS must become a priority for the malaria control policy makers.
